# Discharge performance and dynamic behavior of refuellable zinc-air battery

**DOI:** 10.1038/s41597-019-0178-3

**Published:** 2019-09-09

**Authors:** Woranunt Lao-atiman, Sorin Olaru, Amornchai Arpornwichanop, Soorathep Kheawhom

**Affiliations:** 1 Department of Chemical Engineering, Faculty of Engineering, Chulalongkorn University, Bangkok, Thailand; 2 Laboratory of Signals and Systems, CentraleSupélec, Université Paris-Saclay, Gif-sur-Yvette, France; 3 Computational Process Engineering Research Unit, Chulalongkorn University, Bangkok, Thailand

**Keywords:** Batteries, Energy

## Abstract

Zinc-air batteries (ZABs) are considered a promising energy storage system. A model-based analysis is one of the effective approaches for the study of ZABs. This technique, however, requires reliable discharge data as regards parameter estimation and model validation. This work, therefore, provides the data required for the modeling and simulation of ZABs. Each set of data includes working time, cell voltage, current, capacity, power, energy, and temperature. The data can be divided into three categories: discharge profiles at different constant currents, dynamic behavior at different step changes of discharge current, and dynamic behavior at different random step changes of discharge current. Constant current discharge profile data focus on the evolution of voltage through time. The data of step changes emphasize the dynamic behavior of voltage responding to the change of discharge current. Besides, the data of random step changes are similar to the data of step changes, but the patterns of step changes are random. Such data support the modeling of a zinc-air battery for both theoretical and empirical approaches.

## Abstract (summary)

**Table:** 

Design Type(s)	modeling and simulation objective
Measurement Type(s)	Battery Device
Technology Type(s)	data acquisition system
Factor Type(s)	specific discharge capacity • Electrical Current
Sample Characteristic(s)

Machine-accessible metadata file describing the reported data (ISA-Tab format)

## Background & Summary

Global warming and climate change become aggravated as a result of excessive consumption of fossil fuels. Thus, renewable energy technologies have been actively developed and implemented. Unfortunately, renewable energy sources exhibit inherent intermittent attributes. The innate, erratic nature of renewable energy causes operational difficulties i.e., an unexpected imbalance between energy demand and supply, and lowered power quality. However, the disadvantages of intermittency can be effectively mitigated using an energy storage system^1,2^.

In recent years, metal-air batteries, as a promising energy storage system, have received widespread research interest. As regards the various types of metal-air batteries, zinc-air battery (ZAB) technology shows great potential and is near commercialization.

ZAB exhibits high energy density up to 700 Wh/kg^3^. Zinc is a low-cost metal and is abundant^4,5^. Moreover, it is safe, non-toxic, and environmentally friendly. A zinc-air battery can be fabricated in various designs: namely, a primary cell^6–9^, an electrically rechargeable cell^10,11^, and a mechanically rechargeable or refuellable cell^12–15^. ZAB was commercialized only as a primary cell for lower current application such as in a hearing aid device. For other applications, however, this battery needs to be developed in a variety of facets.

Model-based engineering is one of the effective approaches which can facilitate the development of ZAB. Modeling and simulation can be applied to investigate the phenomena in a battery, monitor its state, optimally operate or assist in designing battery structure^16^. The developed models need to be validated on a variety of real-world configurations. Therefore, reliable experimental data for such validation are necessary. For instance, Mao and White^17^ proposed a primary ZAB model which included precipitation of solid zinc oxide and potassium zincate. Their work used the experimental discharge data provided by MATSI, Inc. to verify the validity of their model. Deiss *et al*.^18^ conducted a discharge and cycle experiment and used the relevant data to verify their own proposed ZAB model. Furthermore, the experimental data was also used to verify the mathematical model proposed by Schröder and Krewer^19^. Recently, Lao-atiman *et al*.^20^ introduced a mathematical model of an integrated system of a zinc-air flow battery and zinc electrolyzer in order to investigate the effect of operating parameters on the efficiency of the system related to hydrogen evolution reaction. The validated data were obtained from the experiment using a homemade zinc-air flow battery and zinc electrolyzer.

Knowledge of ZAB in the model-based engineering aspect is still in the early stages and can be improved considerably. In the aforementioned studies of different battery types, there are missing elements with respect to the ZAB operation, such as dynamic behavior analysis. For example, according to the literature, equivalent circuit models were used to estimate the dynamic behavior of batteries and developed thereby. The equivalent circuit model is an empirical model which can simplify the complexity of the electrochemical model. This type of model often needs low-order approximations to fit the model parameters^21^. Stroe *et al*.^22^ proposed a second order equivalent circuit model and used a current pulse technique to parameterize the dynamic model of a lithium-ion battery. A zinc-nickel battery was also investigated along with the equivalent circuit model^23,24^. The dynamic model was also able to be used to estimate the state of charge of the battery^25,26^. The estimation of the state of charge of the battery is crucial for the application of all types of batteries including ZAB. Both the continuum model and the empirical model require reliable experimental data for validation and parameter estimation.

The purpose of this work is to provide the experimental data for ZAB including discharge profiles at different constant discharge currents, dynamic behavior at different step changes of discharge current, and dynamic behavior at different random step changes of discharge current. All testing data were measured from the home-made tubular ZAB. Discharge profile data were tested within a current density range of 100 mA to 1000 mA. Response data were tested applying various step currents. The data provided can be used to validate the mathematical model of ZAB or estimate the parameters for the empirical model.

## Methods

### Chemical and materials

Nickel (Ni) foams (purity: 99.97%, pores per inch: 100 and thickness: 1 mm) were purchased from Qijing Trading Co., Ltd and were used as cathode current collect. Stainless steel mesh (30 mesh, SUS 304) was purchased from Alikafeii Trading Co., Ltd. and was used as anode current collector and cell chamber structure. Zinc pellets (20 mesh, 99.99% purity) were active material for the battery and were purchased from Sirikul Engineering Ltd., Part. Potassium hydroxide (KOH) pellets (99% purity) purchased from CT Chemical Co., Ltd. was used for preparation of electrolyte. Chemical and materials for preparing cathode consisted of manganese dioxide (MnO_2_, self-synthesized), D-glucose (UNIVAR), acetylene black (AB-50, POLIMAXX, IRPC Public Co., Ltd.), carbon black (BP-2000, Cabot Corporation) and poly(tetrafluoro-ethylene) (PTFE powder, 1 μm, Sigma-Aldrich). Poly (styrene-co-butadiene) was used as a binder. Ethanol and Toluene were used as a solvent. Whatman filter paper No. 4 and poly (vinyl acetate) (24 wt.%, TOA Paint Public Co., Ltd.) were used to prepare the separator. All chemicals were used as received without any further purification.

The synthesis of MnO_2_ was modified from the method published by Pang *et al*.^27^. The aqueous solutions of potassium permanganate (KMnO_4_; prepared from KMnO_4_, UNIVAR) and manganese sulfate (MnSO_4_; prepared from MnSO_4_·H_2_O, QRec) was gently mixed at ambient temperature and pressure. The reaction product was collected by filtration and washed with ethanol. The solid residue was then dried in a vacuum oven at 60 °C and 50 mbar.

### Battery fabrication and operation

**Fig. 1:**
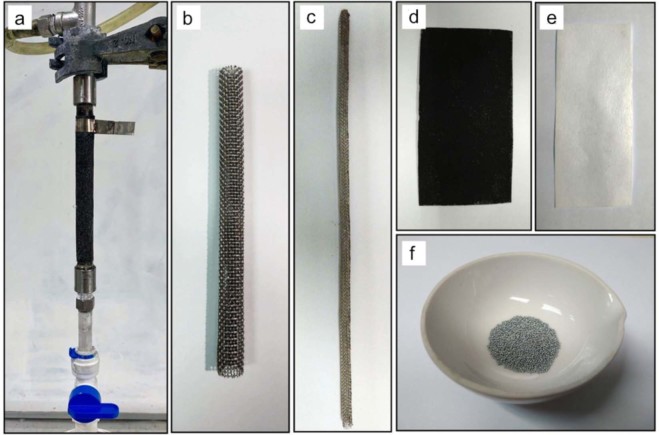
Digital photographic images of a homemade zinc-air battery. (**a**) Fabricated tubular zinc-air battery, (**b**) stainless steel mesh cylinder as a supporting structure, (**c**) stainless-steel mesh tube (the anode current collector), (**d**) the air cathode, (**e**) the separator, and (**f**) zinc pellets used as the anode active material.

Homemade zinc-air batteries were designed and fabricated as a tubular cell, as shown in Fig. 1a. The digital photographic images of each cell component are shown in Fig. 1b–f. The cell structure (supporting structure) was made of stainless-steel mesh rolled into a cylindrical form (Fig. 1b). The cylinder was wrapped with a separator. Whatman filter paper (No.4) coated with 24 wt.% poly (vinyl acetate) solution was used as the separator (Fig. 1e). After that, the cell was covered with the air cathode on the outer layer.

To prepare the air cathode, nickel foam was used as the cathode current collector and substrate for the gas diffusion layer (GDL) and ORR catalyst layer. The active area of the cathode was 29.83 cm^2^. The GDL mixture was composed of 40 wt.% AB-50, 40 wt.% PTFE powder and 20 wt.% glucose. Ethanol was used as a solvent for the GDL mixture. This layer was coated on one-side of the Ni foam and then taken to the hot press for 15 min at 350 °C. After that, the catalyst layer was coated on the other side. The catalyst layer consisted of 70 wt.% BP-2000 carbon black and 30 wt.% manganese oxide. Then, poly (styrene-co-butadiene) was used as a binder, and 5 wt.% of dry-basis catalyst mixture was added. Toluene was used as a solvent for the catalyst mixture. Next, the coated cathode was annealed at 110 °C for 15 min. After annealing, the electrode was pressed by a roll pressing machine until the electrode thickness equaled 1 mm. The finished cathode, as shown in Fig. 1d, had catalyst loading of 3 mg/cm^2^. Finally, after the cathode was finished, it was taken to wrap around the cell by means of facing the catalyst side towards the separator. Before putting the anode inside, the cell was filled with 8 M KOH solution and held for 24 h in order to saturate the separator and be ready for use.

**Fig. 2:**
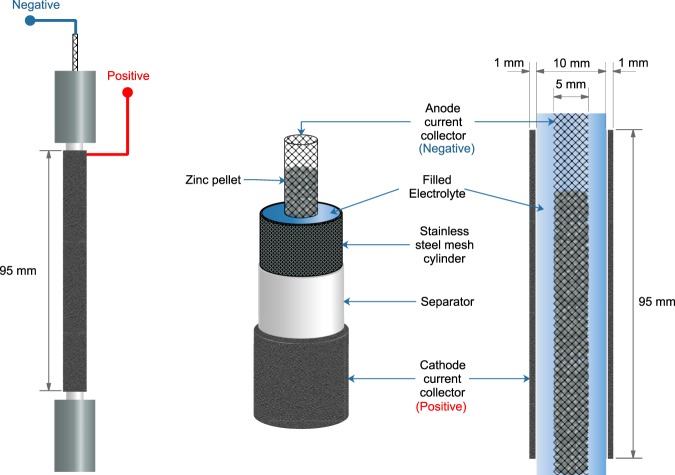
Cell structure and dimension of a homemade zinc-air battery.

The anode was placed in the center of the tubular cell. A 5 mm-diameter tube made of 30 mesh stainless-steel mesh was used as anode current collector. 6 g of 20 mesh zinc pellets were packed inside the current collector tube as anode active material. 8 M KOH solution was used as the electrolyte and was poured into the anode chamber. A summary of cell components and parameters is shown in Tables 1 and 2. The battery was fabricated, as shown in Fig. 2.

**Table 1:** Summary of cell components.

Components	Material
Anode Current Collector	5 mm-diameter tube made of 30 mesh stainless-steel mesh
Cathode current collector	Nickel foam (1 mm thick)
Separator	Whatman filter paper (No. 4) coated with 24 wt.% Poly (vinyl acetate) solution
Anode active material	20 mesh zinc pellets packed inside current collector tube
Cathode active material	Oxygen in the atmospheric air
Gas diffusion layer	Mixture of 40 wt.% AB-50/40 wt.% Polytetrafluoroethylene/20 wt.% Glucose
Catalytic layer	Mixture of 70 wt.% BP-2000/30 wt.% MnO_2_ (catalyst loading of 3 mg/cm^2^) Poly (styrene-co-butadiene) as binder by the amount of 5 wt.% of dry-basis mixture
Electrolyte	8 M Potassium hydroxide solution

**Table 2:** Summary of cell parameters.

Parameters	Values
KOH concentration	8 M
Separator thickness	0.1 mm
Cathode thickness	1 mm
Cathode length	9.5 cm
Cathode active surface area	29.83 cm^2^
Catalyst loading	3 mg cm^−2^
Amount of zinc	6 g
Electrolyte volume	15 cm^3^
Anode current collector diameter (stainless-steel mesh tube)	5 mm
Anode current collector full length	20.5 cm
Zinc pellets bed length (equivalent to 6 g of zinc pellet)	13.5 cm
Anode chamber diameter (stainless-steel mesh cylinder)	10 mm

### Measurement and data collection

After battery fabrication, cell voltage and current were measured by a BA500 battery analyzer using BA500WIN software. The discharge current can be adjusted manually, and cell voltage can be measured at the selected current continuously. Data logging time can also be selected. The output files of the collecting data were.csv files. The data were rearranged, and the file type was changed into.xlsx. The data file contains various information, including working time, cell voltage, discharge current, discharge capacity, discharge power, discharge energy, and temperature. A discharge profile test was executed by discharging the battery at selected values of discharge current and measuring the evolution of cell voltage until the battery was exhausted. The measurement of discharge profile test was terminated when the discharge current was lower than the setpoint current. As for the step discharge test, the voltage was measured when the discharge current was step-changed. The random discharge test is similar to the step discharge test except that the patterns of the step changes are more random in each test. Data logging time was 1 sec for the step and random discharge test and 5 sec for the discharge profile test.

## Data Records

The data provided can be divided into three categories: discharge profiles at different constant discharge currents, dynamic behavior at different step changes of discharge current, and dynamic behavior at different random step changes of discharge current. Three dataset categories can be separately found in the DischargeProfiles.xlsx, RandomDischarge.xlsx, and StepDischarge.xlsx. These datasets are available at the repositories^28^. In Table 3, metadata which provide the description for each data column are presented.

**Table 3:** Metadata of discharge and response test.

Data	Unit	Description
Total time	S	Total operating time
Voltage	V	Measured voltage of the battery
Current	mA	Measured current of the battery
Result	mAh	Calculated capacity of the battery
Power	W	Calculated power of the battery
Energy	Wh	Calculated energy of the battery
Temp	°C	Room temperature

## Technical Validation

In the experiment, data were collected from different batteries. Every time a new test was carried out, the anode and electrolyte were changed. Thereby, a deviation in the data set was apparent and affected battery capacity. It represents a usual phenomenon which should be accounted for in future large mass production. Besides, the deviation might also have affected the voltage, but this was less than the effect on the capacity. For all data, the erroneous data point of voltage was removed and smoothed. It was noted that such incorrect data occurred at a lower current range or near the OCV range. Such errors caused the voltage values to be higher than the real values. The method used to remove the erroneous data is the interpolation between non-error points.

**Fig. 3:**
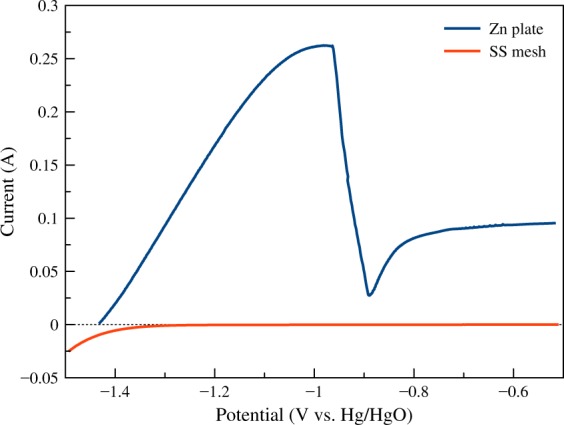
Linear sweep voltammograms of the zinc plate (scanned from OCV to −0.5 V vs. Hg/HgO), and stainless-steel mesh (scanned from −1.5 V to −0.5 V vs. Hg/HgO) with a scan rate of 5 mV/s.

To check the electrochemical compatibility of the materials used in the battery, the electrochemical characteristic was examined by linear sweep voltammetry (LSV). The LSV was performed by a potentiostat/galvanostat (AMETEK, VersaSTAT 3). Three electrodes configuration was used with platinum as the counter electrode and mercury/mercury oxide (Hg/HgO) as the reference electrode. The electrolyte used in all tests was 8 M KOH solution. The linear sweep voltammograms of the zinc plate and stainless-steel mesh are depicted in Fig. 3. The dimension of zinc plate was 1 × 1 cm^2^. The test of the zinc plate scanned from the open circuit voltage (OCV) to −0.5 V vs. Hg/HgO with a scan rate of 5 mV/s. The OCV of zinc plate was 1.44 V vs. Hg/HgO. As the potential scanning proceeded, the current positively increased, which is known as oxidation current. The oxidation of zinc increased until reaching the peak at −0.98 V vs. Hg/HgO. Scanning further caused decreasing in current due to the passivation of zinc surface, and the depletion of hydroxide ion at the surface of the electrode^29–31^. Because stainless-steel mesh was used as the anode current collector, the electrochemical characteristic of stainless-steel mesh has to be checked in the same potential range as the zinc. The dimension of stainless-steel mesh was also 1 × 1 cm^2^. The stainless-steel mesh was scanned from −1.5 to −0.5 V vs. Hg/HgO with a scan rate of 5 mV/s. The small negative current was detected at the potential about −1.5 to −1.3 V vs. Hg/HgO. The negative current is the reduction reaction, which is contributed to a hydrogen evolution reaction. Nonetheless, the potential range of −1.2 to −0.5 V vs. Hg/HgO showed almost no current. It means that stainless-steel mesh was stable and did not oxidize in this potential window. The result showed that the zinc is compatible with stainless-steel mesh in the operation potential of the battery. At the OCV, the stainless-steel mesh conducted a small amount of hydrogen evolution, which can promote corrosion of zinc. However, it was observed that the hydrogen evolution current of stainless-steel mesh was minimal compared with the zinc oxidation current.

**Fig. 4:**
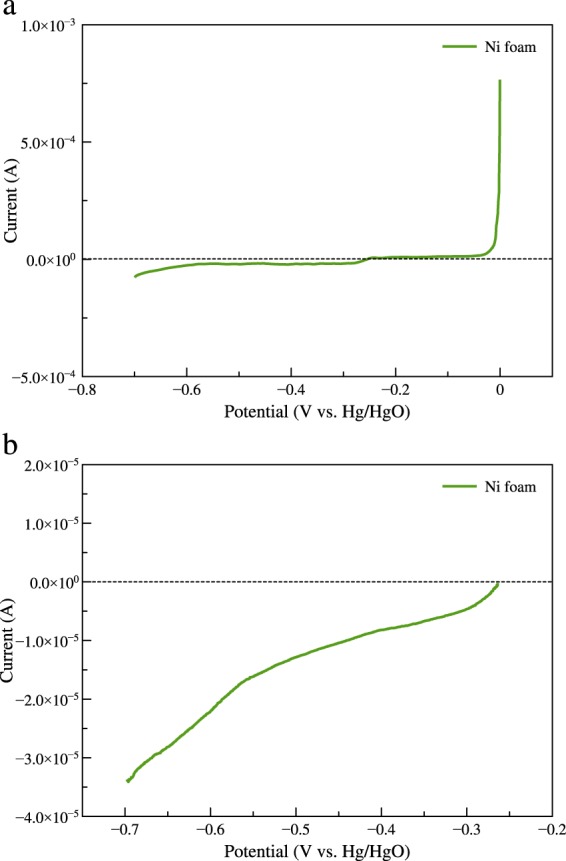
Linear sweep voltammogram of the Ni foam with scan rate of 5 mV/s. (**a**) Scanned from 0 to −0.7 V vs. Hg/HgO, and (**b**) scanned from OCV to −0.7 V vs. Hg/HgO.

Because nickel foam was used as the cathode current collector, the electrochemical characterization in the potential range of air cathode reaction must be checked. The LSV of nickel foam is presented in Fig. 4. As regards Fig. 4a, the 1 × 1 cm^2^ nickel foam was scanned from 0 to −0.7 V vs. Hg/HgO with a scan rate of 5 mV/s. Before the test, the electrolyte was purged with nitrogen gas for 30 min in order to reduce the effect of dissolved oxygen. Small oxidation current was noticed at the potential range of 0 to about −0.25 V vs. Hg/HgO. This oxidation current might come from the oxidation of nickel. At the potential range of −0.25 to −0.7 V vs. Hg/HgO, small reduction current was observed. The reduction current might come from the reduction of remain dissolved oxygen in the electrolyte and the nickel oxide on the nickel surface. To focus on the reduction reaction, nickel foam was scanned from OCV to −0.7 V vs. Hg/HgO, as shown in Fig. 4b. It was found that the order of magnitude of the current of nickel foam was very low. It can be inferred that the reduction of nickel metal slightly occurred in the test potential range. Therefore, the reaction of nickel metal does not interfere the oxygen reduction reaction of the air cathode.

## Usage Notes

The data provided herein has been useful in assisting model-based engineering for a zinc-air battery. Thus, the data can be employed to validate the result of the theoretical model or fit the parameters of the empirical model. It should be noted that this data was collected from the home-made battery, as shown in Fig. 1. For this type of cell structure, battery behaviour, and phenomena might be unique. It is difficult to compare the data as mentioned above to the data as measured from the batteries having different structures.

## ISA-Tab metadata file

Download metadata file

## Data Availability

The reported data were generated form experiments, and not relevant to any computer codes.
